# New Jersey State Dental Society

**Published:** 1896-11

**Authors:** 


ARTICLE II.

### NEW JERSEY STATE DENTAL SOCIETY.

The twenty-sixth annual meeting of the New Jersey State Dental Society was held at Asbury Park, N. J., July 29th, 30th, and 31st, 1896.

JULY 29TH, MORNING SESSION.

The meeting was called to order by the Vice-President, Dr. Harvey Iredell, the President being absent. The session was opened with prayer by the Rev. Joseph G. Reed. The chairman then explained that the President, Dr. Sanger, had unexpectedly gone to Europe, but had left his address, which was then read by the Secretary.

### ANNUAL ADDRESS BY THE PRESIDENT, R. M. SANGER, D. D. S.

To-day begins the twenty-sixth year of our existence. With the closing of the first quarter of a century, we have passed through our childhood and youth, and stand in the full strength and vigor of manhood with all the hope and promise of a brilliant future. The promise is strengthened as we take a retrospective view of our history, and contemplate, with pardonable pride, the work aecomplished and the strides made in the elevation of our profession in this State. Especially is this true along the lines of a higher standard of dental circulation, where you have always been found in the forefront of the battle; but that all is not yet accomplished is shown by the results of the work of your Legislation Committee this winter.

Having prepared an amendment to the present State Dental Law, which made it as nearly perfect as it is possible to make any general State Law; and having successfully carried it through the Senate, it was finally sidetracked in the House, through the political influence of a man, who is able to practice in this State to-day because of a lack of funds wherewith this Society could properly prosecute him. It is an open secret that if your committee had consented to grant this man immunity from prosecution under the law, all opposition to the passage of the bill would have been withdrawn; and I congratulate you on the possession of a committee, whose good judgment and high sense of honor made them stand firmly on the ground that honorable defeat is preferable to dishonorable victory. This brings us face to face with a weak point in our armor, which we should now look to the Legislature to repair. The remedy lies within ourselves. Ever since the first Dental Law was passed we have been hampered by a lack of funds with which to get the cases of infraction of law in such shape that they could be presented to the court with a reasonable hope of a favorable decision; and while many have given up illegal practice, rather than face the prospect of a law-suit, some few have discovered our weakness, and have grown bold, and openly defying us, as in the case just cited. I would, therefore, recommend the establishment of a "Prosecuting Fund," the collection and disbursement of which shall be in the hands of a committee to be appointed during this meeting in such manner as you may deem best.

In National as well as State dental education, New Jersey has always played an important part, and it is not surprising to find among the best of officers of the National Board, the name of our esteemed member, Dr. Charles A. Meeker, as Secretary.

Having served us most acceptably, for 21 years, in a similar position, it is not strange that immediately upon his becoming a member of the National Board, they should have sought his services in this important office. This is the natural result of a well-earned social and professional reputation. Our Society having gained National fame, his reputation as your Secretary has also become National.

To find a single name missing from the roll has been an unusual experience for this Society in recent years, consequently it is with extreme sadness that this year we are called upon to mourn the loss of one of our members—Dr. H. Lyman Clarke, of Rahway, and I recommend the appointment of a committee to draw suitable resolutions touching the loss of our brother.

Of the work of the various standing committees, too much cannot be said in praise. Every department shows unmistakable evidence of vigor and push, which is the best proof of the healthy condition of the Association. But, as others learn from us, so must we learn from them; and I would respectfully call the attention of the Committee on Materia Medica to the anvanced work being done by the similar committee of the State of Illinois. In this connection, I would recommend the formation of a committee to investigate and report upon the advisability of establishing a chemical laboratory, under the supervision of this society.

The programme which is before you is a sufficient guarantee of the good things that await you, and I will not detain you longer, only pausing to compliment the committee on its very efficient work, and to say to our visiting friends that the hand of welcome is gladly extended to them; and that the full privileges of the floor is theirs as long as they remain our guests.

Upon motion of Dr. G. Carleton Brown, the address of the President was referred to a committee of three, who should report upon the adoption of the suggestion recommended.

Secretary Meeker reported that he had brought with him the minute book containing the proceedings of the Society at its last meeting, and would read the minutes if the members desired. He had made a contract with the S. S. White Company, by which they agreed to have the proceedings of the Society published prior to the present meeting, but that they had failed to do so.

The reading of the minutes was passed.

Upon motion of Dr. G. Carleton Brown, a committee of three was appointed to confer with other journals and arrange terms of publication of the proceedings of the meeting of 1896.

Dr. Meeker read a letter from Dr. Donnelly requesting the Society to co-operate with the American Dental Association in the work of sending dental material to the National Dental Museum and Library at Washington.

Dr. L. Ashley Faught reported that he had personally visited the Museum and found therein already a wonderful collection. Space has been set aside to receive any collections which may be sent, and they are willing to purchase everything which will be of value. He thought that a committee should be appointed to carry out Dr. Donnelly's wishes.

A motion prevailed to this effect.

The Secretary, Dr. Meeker, read a letter from Dr. J. Milton Smith, apologizing for some references in his paper of the year before.

### AFTERNOON SESSION.

The Vice-President, Dr. Iredell, introduced Dr. L. Ashley Faught, of Philadelphia, who read the following paper:

### ORAL HYGIENE.

Nearly ten years ago, it was my privilege to read a paper on this subject before the Pennsylvania State Dental Society. The germ of what I said at that time was: That Oral Hygiene is the work of the patient, and comprises the preservation and maintenance of the dental organs in a condition of health. At the same time I formulated for the profession a new creed. "Failure in operations and tooth loss are mainly due to the lack of oral hygiene." Ten years have only served to confirm this dogma.

There is nothing more gratifying to the conscientious dentist than the knowledge that his efforts are intelligently seconded by his patients. Our duty is, therefore, to advise and instruct our patients generally, or in accordance with the necessities of special conditions, but we are not called upon to turn our offices into dispensing drug-stores, or into depots for the *sale* of tooth-washes, tooth-brushes, tooth-powders, floss silk, etc. The plea of accommodation is not adequate in large cities, where the drug-store is adjacent to almost every home. Nothing in this stricture is to apply to our handling to a patient, as a gift, a sample of that which we advise; for I find that where this is done, the patient more promptly and thoroughly follows out our professional advice.

The time when the use of a tooth-brush alone, or perhaps loaded with a dentifrice, was accepted as all-sufficient on the part of the patient, has well-nigh passed. More is now to be expected and advised. It is true that these valuable adjuncts cannot be laid aside or ignored, but their use is to be supplemented.

At the risk of having this paper charged with being elementary, it is proposed to consider tooth-brushes, tooth-powders, tooth-washes and mouth-washes.

First of all there is the tooth-brush. The combined wisdom of the profession has made its impress on this article, and manufacturers have endeavored to follow our teaching; still the forms and shapes in use are numberless —few combining all the good points. My own ideal is the "Prophylactic," and I believe it to have the acceptance of most of my professional brethren. Through my advice, it is in almost universal use among my patients. If I am wrong in my selection, I shall be most happy to correct the error; but what I deem important is that, as a profession, we should agree upon and universally advise the same. My argument is that there is a correct shape, and that we as dentists should decide upon it finally so that recommendation to our patients would prevent the use of any other, except where a deviation was made for specific purposes.

It is absurd in this age to say: Any kind, so it is a brush. You might just as well say: Use it in any manner. The mode of use not long ago became a matter of accepted teaching, and I find upon inquiry, that few people to-day are unaware of it. The large majority have been properly instructed. Let us now begin at the beginning—the construction of the brush itself. That is the foundation of the first step in oral hygiene.

Tooth-powders and tooth-washes do not demand that they should be so specifically considered. Unanimity regarding their composition and use has practically existed for years, and no radical departure now seems to be required.

Mouth washes being more in the nature of a medicament have received at the hands of the profession more general consideration, and it would be assumption on my part to take up your time by discussing those already well known. There is, however, one which is comparatively new, and with which I have during the last year experimented considerably with much satisfaction—Borolyptol.

When my attention was first directed to this preparation, I looked upon it as only one of the many others in the market, akin to the well-known Listerine. It is quite different.

The formula given is:

Formaldehyde 0.2 per cent.
Aceto-boro-glyceride 5 per cent.
With Pinus pumilio,
Eucalyptus,
Myrrh,
Storax,
Benzoin.

Two principal ingredients, and five which may be looked upon as flavoring matters. The benefits obtained are due to the two, and have been said to be due to the one, Formaldehyde. I am inclined to believe they are due to the two, and for the following reasons:

Some time ago, in the latter part of 1883 and the spring of 1884, my attention was directed to a preparation which was then introduced to the medical profession by my brother, G. Granville Faught, M. D., which he called Boroglyceride. It was essentially a combination of Boracic acid and Glycerine, of a light amber color, vitreous, and without odor, soluble in glycerine in all proportions, and by applying heat, to the extent of 10 per cent. in water. From the very nature of the ingredients it was non-irritating, soothing, detergent, astringent and antiseptic.

I made considerable use of this medicament, which proved itself non-toxic and non irritant to the tissues. The Aceto-Boro-glyceride in Borolyptol is the embodiment of these features in more convenient form.

In the Ephmeris of Dr. Squibbs for 1884 and 1885, Vol. II, p. 796, the effects of Boric acid are referred to when used as a protective to prevent vegetable growths in other fluids, and a series of experiments extending over six months given, with results showing "a strong probability that Boric acid in the proportion adopted—namely, a half of one per cent.—will protect these solutions until they are used up in any ordinary practice."

A fluid, then, made up alone of five such ingredients as Pinus, Pumilio, Eucalyptus, Myrrh, Storax and Benzoin, approximates Listerine in its nature and uses, and commends itself as a mouth-wash to the dental profession. When, however; Aceto-Boro-glycerine is added, it is, by the addition of the qualities we have just studied, moved forward into a much higher class; and when we go still further and add Formaldehyde, we carry it beyond the others into a class by itself, for it has supplied to it then a germicidal quality, without the addition of anything poisonous or irritating.

In substantiation I quote Dr. J. C. Smith, Director New York Post-Graduate Medical School Laboratory, who says: "Borolyptol is equal to, and in some instances a better germicide than a 1-1000 Bichloride of Mercury solution," which testimony has also been made by Dr. Philip Jaisohn, Pathologist in the Garfield Memorial Hospital, Washington, D. C.

The effects of Formaldehyde in a 40 per cent. solution (Formalin) have been abundantly proven to be far more efficient in much smaller quantities than Corrosive Sublimate, Borax, Boric acid, Salicylic acid or Benzoic acid: and exceedingly potent when used in even less proportion.

Two English practitioners of medicine, Mr. Charles Slater, M. R. C. S., and Mr. S. Rideal, D. Sc., both of London, England, have carried on a complete series of experiments, aud find it possesses very decided antiseptic and disinfectant properties. Those interested are referred to their report on "Formaldehyde as an Antiseptic," in the London *Lancet*, Fol. I; for 1894, p. 1004.

The impression on my mind made by this knowledge regarding Boroglyceride and Formaldehyde led me to make use of Borolyptol and recommend it to my patients as a mouth-wash, either dilute, or in full strength. I have found in its application a substance that may prove exceedingly useful in the hands of the dental profession; it is certainly worthy of your attention and investigation, and makes a forcible plea for its admission into the dental materia medica.

### DISCUSSION.

Dr. C. S. Stockton.—If there is one thing that is lacking in the dental profession to-day, it is not the ability to fill teeth, not the ability to supply the deficiency when the natural teeth are lost, but it is in the care with which they pretend to cleanse the teeth.

I have seen a great many patients who have had splendid work done; none could be better, so far as the fillings were concerned. They were an honor to the gentlemen who did them, yet all around, under the margins of the gums, were the accumulations not only of a year, but of years. Unless intelligence is used in cleansing the teeth, you may use Borolyptol with no better effect than with water. Nor is there any excuse for this after all that has been said and written, and with the excellent instruments that are at your disposal. No patient should leave your chair unless the teeth are thoroughly cleansed.

My friend, Dr. Faught, has gone a good way in his recommendation of Borolyptol. It may be all right—I don't doubt that it is—but our friend, Dr. MacKellops, has so hammered at the profession that I did not suppose there was any man to-day brave enough to lend his name to any patent medicine, as Dr. Faught has. MacKellops thinks (I am not saying what I think) that it is degrading the profession, and he has a record of every man who has done anything of that kind, and you will notice that, since the scourging of our friend Mac, very few men have the courage to recommend these preparations.

Now as to tooth-brushes, I understand there is only one firm that makes the Prophylactic brushes. I would not like to go as far as some; but, eliminating that brush from the discussion, nine-tenths of the brushes on the market to-day are not fit to be used! They are entirely too large. Some of these large brown brushes are not even fit to go in a horse's mouth—it would be an insult to a horse to attempt to brush his teeth with one.

There is another thing which many of us fail to impress upon our patient, and that is, that the gums need brushing as well as the teeth. If the patient will do that properly it matters little whether he uses Borolyptol or water.

Dr. J. Foster Flagg, Philadelphia, Pa.—It was only recently that the Academy of Stomatology, of San Francisco, had presented to it a most exhaustive *resume* on bacteriological work in regard to oral matters. The paper by Dr. Younger was drawn from reliable sources, and was a very excellent one. But what was the discussion which ensued? After this elegantly presented paper had been read one gentleman said "* * * had been very much interested in this subject. It was a subject upon which he knew little or nothing; that he recognized the importance of keeping a dental office clean, and he thought it was a very important thing that the dentist should keep his finger nails clean." Another gentleman arose and said that, like his predecessor, he " * * * had very little if any knowledge upon this important subject; but he also felt that it was very important indeed that cleanliness should be urged upon the dental profession." Such a dirty, miserable crowd as they generally are, he thought the importance of cleanliness should be urged upon them. He said he was often impressed by the remarks of patients, that Dr. So and So was clean and his office neat and they always liked to go to him, and that a certain other Doctor's office was dirty and they did not like to go there. But what impressed him most of all was that the dentist should keep his finger nails clean! The third gentleman who discussed the paper said that he was very much impressed with the paper and he thought it should be impressed upon the dental profefession not only to be cleanly about their office and instruments and surroundings, but also about their persons and clothes.

And that is all the discussion accorded to that admirable paper by the learned members of the Academy of Stomatology, of San Francisco. Moreover, that is a typical discussion of a paper read before a dental society. If you read the discussions throughout the United States, you will find that they are just about as scientific.

The subject now before us, as our friend has presented it, it seems to me is a very important matter. The hygiene of the mouth has very nearly all to do with the success of our work, that is, the maintenance of the teeth *in situ*. We recognize that although our friend, Dr. Black, has said that there is no choice in filling materials, he said that because he did not know anything at all about it. We recognize certain teeth which we can fill with gold, and when the patient asks us how long it will last, we tell him that we will see him at the end of the first forty years and then talk to him about it. I know the Good Book says that we know not what a day or an hour may bring forth, but we recognize that, other things being equal, that filling will be there thirty to forty years from that day, whether the patient be dead or alive. Further than that, we also recognize that if we fill another class of teeth with gold, at the end of forty years they will not be there at all.

I regard this paper as a first-rate advertisement of what I believe to be a first-rate article.

I suppose Faught is not afraid of Mac—I know I am not!

Under these circumstances, and as I have never seen any particular reason for antagonizing advertising, I will say that if a man feels conscientiously—more than that, if he knows scientificially that a material is a good one—I do not see why he should not say so.

I do not take much stock in tooth-brushes. My friend, Dr. Lord, of New York, on one occasion said that he was not sure—and he is a man of a great deal of observation and experience—but that it would have been better for the teeth if all the tooth-brushes had been at the bottom of the Red Sea. I have always believed that anything which Dr. Stockton says about dentistry is true, and as he says that nine-tenths of the tooth-brushes are good for nothing, then I think Dr. Lord's opinion is about my opinion. I think that ninety-nine one hundredths of the tooth-brushes sold in the drug-stores are good for nothing. Then shape does not amount to anything, and you know shape is a great thing! Their bristles do not amount to anything—they are altogether too stiff or altogether not stiff enough. A happy medium in stiffness is the great thing under all circumstances. It seems to me that the subject of brushes ought to be ventilated at just such a meeting as this.

But when it comes to the scaling of teeth, the removal of something which requires instruments, let me tell you that there is not a scaler in a thousand that is worth a dollar a thousand! The only scalers that have been of any service in my hands are modifications of the Riggs instruments, and you will not find one sold over the counter of any dental depot in this world that amounts to anything, unless it is specially ordered. My friend Jardic, of the University of Pennsylvania, on one occasion remarked that it is self-evident that they are good for nothing. I am not sure whether it was Jardic or Barker, it was one or the other—Barker is dead, so he cannot make any denial.

Now, it seems to me, that when it is self-evident that a thing is good for nothing, it is worth investigation; for things that are self-evident I have strong doubt about. So I obtained from Jefferson Riggs a set of scalers; and while they were awkward to use in the beginning, still, after a few months of persistent use, I found they were certainly instruments with which I could do practically everything in the scaling of teeth.

Only a short time ago one of our demonstrators came into the clinic with a case in which he said he had done all that he could with two lower incisors, and that the patient must have constitutional treatment. With delicate probes I detected small excrescences on the lower portion of the teeth, and I said, "Dr. Hughes, these teeth only require scaling." "Why," he said, "they have been scaled." I took one of Riggs' scalers, straight as a bayonet—the bayonet is the thing to rely upon when you want to make a fight—the bayonet is the thing that tickles! I passed it down and said, "Listen—click! click! click! And these are the teeth that have been scaled!" But when they had been scaled, and the mouth carefully rinsed out with not too much Borolyptol, the trick was done, and a few days later my young friend Hughes presented himself at the clinic and reported that everything was nice as pie—apple pie!—and I told him that all the constitutional treatment needed was the point of a "constitutional" scaler!

But this subject is perfectly exhaustless, it should be discussed all this afternoon and evening, and all the time until the officers are elected.

This is my idea of the subject of "Oral Hygiene."

Dr. Faught.—If anything I have presented in this paper is an advertisement, I care nothing for that part of it. I am one of those who rarely puts his name to a paper which he does not believe contains facts which he believes to be true. What I have said in this paper I believe to be true, and I subscribe to it.

I suppose that every gentleman here who knows me would recognize that he had never—I say "never"—seen my name attached to an advertising circular concerning an instrument, and I hope he never will. But I distinguish between writing such things and stating facts before an association of this kind.

There is perhaps no preparation which has been so much pushed as has Borolyptol, and if any of you gentlemen, who have the facilities, will take the trouble to look into your libraries, or into libraries that you may have access to, you will be surprised to find how little you can discover upon the subject of Borolyptol. It was because I had some knowledge upon that subject, because of my work in connection with Boroglyceride, that I deemed it my duty, in presenting this subject, to deal with Borolyptol in such a way that it would put you upon the track of the literature referred to.

More than that. The course of the medical profession has been such that its members have gone out of their way to use remedies that would not do the work one half as well as preparations put up for them by manufacturing chemists, with the formula attached. In the present case I dealt with the formula and nothing else. I felt we were dealing with a thing we ought to know more about, and I have presented this paper as a scientific contribution to a scientific body, for scientific discussion.

Dr. George S. Weld, New York.—I want to say just a word. Take for instance various dental preparations on the market which are composed of nothing but alcohol and essential oils. Possibly they may be classed as patent preparations. But it seems to me that any preparation which, like Borolyptol, gives the formula upon the label, is not a patent affair. It may be a proprietary preparation, and possibly some here may be interested in it. I do not think for a moment, however, that Dr. Faught is financially interested in any proprietary preparations. But if a reputable house, with superior facilities, puts up something possessing merit in convenient form and in a scientific manner to be used by dentists, it seems very proper to mention it in a paper such as Dr. Faught has read, without laying the writer open to a charge of advertising. Furthermore, it is a legitimate subject for discussion.

I hold in my hand a bottle of Euthymol, which is a somewhat similar preparation to Borolyptol. I have no financial interest in it; but if I find it to be a good thing I shall feel it quite proper to state it before this or any other society.

The formula is given upon the label.—*Items of Interest*

---

ARTICLE III.

